# Warfarin and boceprevir interaction causing subtherapeutic international normalized ratio: a case report

**DOI:** 10.1186/1752-1947-8-433

**Published:** 2014-12-17

**Authors:** Andrew S Tsiattalos, Anita Patel

**Affiliations:** 21 Clinical Pharmacist, Critical Care, VA New Jersey Healthcare System, East Orange, NJ USA; 22 Clinic for Anticoagulation, Philadelphia VA Medical Center, Clinical Pharmacy Specialist, Philadelphia, PA USA

**Keywords:** HCV, Hepatitis C virus, HCC, Hepatocellular carcinoma, INR, International normalized ratio

## Abstract

**Introduction:**

Chronic hepatitis C is a leading cause of severe liver disease. Protease inhibitors used to treat these patients are known to have many drug interactions, although there is limited data available between boceprevir and warfarin. This case report is the first *in vivo* drug interaction reported in the literature.

**Case presentation:**

A 73-year-old African American man was diagnosed with hepatitis C in 2004, and had decided to not initiate therapy. In 2006, he was diagnosed with deep vein thrombosis and pulmonary embolism and was started on warfarin. His international normalized ratio had been stable on a dose of 13.75mg to 20mg/week over a period of 6 years. A liver biopsy in 2012 revealed marked fibrosis, leading the patient to start hepatitis C treatment with peginterferon alfa-2a, ribavirin and boceprevir. Three weeks after starting boceprevir, his international normalized ratio became subtherapeutic at 1.2. Upon increasing the warfarin dose by 16%, his international normalized ratio remained at 1.2 6 days later. Two months after initiating boceprevir, he reached a therapeutic international normalized ratio. His warfarin dose had been increased by 75% from his dose prior to starting boceprevir, from 15mg/week to 26.25mg/week. His hepatitis C treatment was discontinued at week 39 of the intended 48 weeks of treatment due to severe thrombocytopenia. Upon discontinuation of boceprevir, his warfarin dose was prophylactically decreased by 17%, which resulted in a subtherapeutic international normalized ratio of 1.48 1 week later. The warfarin dose was subsequently increased by 10% which resulted, 2 weeks later, in a therapeutic international normalized ratio of 2.8. Once stabilized, his new warfarin dose was 23.75mg/week, 37% higher than his original maintenance dose of 15mg/week prior to starting boceprevir.

**Conclusions:**

The co-administration of boceprevir and warfarin resulted in a subtherapeutic international normalized ratio. Upon starting boceprevir, his warfarin dose was increased by 75% over 2 months to achieve a therapeutic international normalized ratio. After discontinuing boceprevir, his maintenance dose of warfarin was 37% greater than his original dose. This is an original case report which demonstrates the significant effects of this drug interaction and the importance of monitoring international normalized ratio.

## Introduction

Chronic hepatitis C virus (HCV) is an infection that affects approximately 2.7 to 3.9 million people in the USA with an estimated mortality rate of 12,000 deaths per year according to the US Centers for Disease Control and Prevention [1]. HCV is a progressive disease and a leading cause of cirrhosis, end-stage liver disease, hepatocellular carcinoma (HCC), and liver transplantation. In acute cases of HCV infection, 75 to 85% will become persistent, of which 20% will develop cirrhosis after approximately 20-30 years [1].

There are reportedly six different genotypes of HCV in the world, with genotype 1 being the most common in the USA [1]. With the advent of HCV protease inhibitors in addition to the standard regimen of pegylated interferon and ribavirin, sustained virological response rates in patients with chronic HCV genotype 1 have improved from nearly 40 to 50% to approximately 60 to 80% [2].

Boceprevir is a protease inhibitor indicated for the treatment of chronic HCV genotype 1. The mechanism of action is a selective inhibition of the HCV NS3/4A serine protease [3]. Dosing is 800mg every 8 hours, and the duration of triple therapy with peginterferon and ribavirin is dependent on the patient’s response to treatment, which can vary between 24 to 48 weeks of treatment [2, 3]. There are many drug–drug interactions with boceprevir, although to the best of our knowledge there have not been any documented reports of an interaction with warfarin. Here we present a probable interaction between boceprevir and warfarin.

## Case presentation

The patient is a 73-year-old, 79kg African American man with a past medical history of diabetes, hypertension, and microscopic hematuria. He was diagnosed with HCV genotype 1a infection in 2004. At that time, he was not interested in being treated with interferon and ribavirin given poor response rates and profound adverse effects. In June 2006, he was diagnosed with deep vein thrombosis and pulmonary embolism, for which he was started on warfarin and referred to the anticoagulation clinic for drug monitoring. The duration of warfarin therapy was determined to be indefinite by his primary care team. His target international normalized ratio (INR) is 2 to 3, which had been stable on a warfarin dose of 13.75mg to 20mg/week, over a period of 6 years.

He underwent a transjugular liver biopsy in April 2012 due to possible cirrhosis and for guidance of possible HCV treatment. The biopsy showed mild fatty infiltration of his liver and received a grade of stage F4 with marked bridging fibrosis. At this time, he was interested in HCV treatment. It was decided that he would begin treatment with peginterferon alfa-2a weekly and ribavirin twice daily for the first 4 weeks. Boceprevir would be added at week five to be given every 8 hours for 44 weeks. He was informed that treatment could be discontinued if he did not demonstrate a positive and safe response to therapy. Counseling on medication adverse effects and required laboratory monitoring were reviewed with him.

HCV therapy with peginterferon alfa-2a and ribavirin began on 1 August 2012. At this time, his INR was stable on a weekly warfarin dose of 15mg for the previous 4 months. Boceprevir was scheduled to start on 29 August, although he started it on 31 August. On the day prior to starting boceprevir, his INR was 2.5. Five days after starting boceprevir, his INR remained therapeutic at 2.5. On 19 September, almost 3 weeks later, his INR decreased to 1.2. He denied any missed doses of warfarin or changes in consumption of vitamin K foods. His warfarin dose was increased by 16% and 6 days later, on 25 September, his INR remained subtherapeutic at 1.2. However, the day before this visit, he had eaten a large portion of collard greens, a high vitamin K food. The anticoagulation provider believed this may have contributed to the low INR. Therefore, the provider instructed the patient to take a booster dose and remain on the same weekly dose of 17.5mg/week. At his following week’s appointment, his INR was still subtherapeutic at 1.3 without any vitamin K intake or missed doses of warfarin reported. He continued to come to the anticoagulation clinic every 7 to 10 days for INR monitoring and subsequent dose increases; see Table 1 for more details. The majority of his INRs were checked on a point-of-care (POC) device. Periodically, his INRs were also checked in the laboratory through a venipuncture blood draw. On two occasions, both a POC and a laboratory INR were taken on the same day and results correlated very well with one another.

**Table 1:** **Warfarin dosing relative to initiation and discontinuation of boceprevir**

Date	Weekly ribavirin dose (mg)	Weekly warfarin dose (mg)	POC-INR, LAB-INR*	Important notes
11/07/2012		15	1.9	
01/08/2012	1200	15	2.8	PEG-INF and RBV started
15/08/2012	1200	15	1.9	
30/08/2012	1200	15	2.5	
31/08/2012	1200	-----	-----	Boceprevir started
05/09/2012	1200	15	2.5	
19/09/2012	1000	15	1.2	
25/09/2012	1000	17.5	1.2	Consumed high vitamin K food on 24/09/12
01/10/2012	600	17.5	1.3	Epoetin alfa and vitamin B12 added
10/10/2012	600	20	1.3	
16/10/2012	200	23.75	1.48*	
24/10/2012	200	26.25	2.2	
31/10/2012	200	26.25	1.72*	
07/11/2012	200	27.5	2.0	
14/11/2012	200	26.25	1.8 1.82*	Patient took less warfarin than instructed.
27/11/2012	400	27.5	2.24*	
12/12/2012	600	27.5	2.0 2.02*	
26/12/2012	800	28.75	2.1	
16/01/2013	800	28.75	1.7	
30/01/2013	800	31.25	2.0	Consumed high vitamin K food twice in past week
13/02/2013	600	31.25	3.3	
27/02/2013	200	30	2.4	Iron added
13/03/2013	400	30	2.9	Recent suprapubic abscess; completing amoxicillin/clavulanate, which started 07/03/13
27/03/2013	200	30	2.2	
10/04/2013	200	30	2.4	
24/04/2013	400	30	3.6	New facial abscess, starting 10-day course clindamycin
08/05/2013	400	28.75	2.5	HCV medications DISCONTINUED due to low platelets
16/05/2013		23.75	1.48*	
29/05/2013		26.25	2.8	
12/06/2013		Unknown	2.2	Hospitalized at outside hospital for UTI and bacteremia from 04/06/–07/06/13, ciprofloxacin added
19/06/2013		25	3.1	
26/06/2013		25	3.7	
10/07/2013		25	3.2	Patient took more warfarin than instructed
24/07/2013		23.25	2.4	
15/08/2013		23.75	2.4	
10/09/2013		23.75	3.0	
10/10/2013		23.75	2.5	
06/11/2013		23.75	3.2	

*Abbreviations:* *HCV* hepatitis C virus, *INR* international normalized ratio, *LAB* laboratory, *PEG-INF* peginterferon alfa-2a, *POC* point-of-care, *RBV* ribavirin, *UTI* urinary tract infection. *Laboratory INR.

He finally reached a therapeutic INR on 24 October 2012, almost 2 months after starting boceprevir. His original warfarin dose was increased by 75% from 15mg/week to 26.25mg/week. In the subsequent weeks, his INR trended downward due to an increased consumption of vitamin K foods, for which his dose was further increased to 30mg/week. At this point, his original dose had doubled in order to maintain a therapeutic INR. For approximately 2 months thereafter, he remained stable on 30mg/week of warfarin. On 24 April 2013, his INR increased to 3.6 while on 30mg/week of warfarin probably due to a decrease in vitamin K food intake. At this point, his warfarin dose was reduced and his INR returned to his therapeutic target by his next anticoagulation clinic visit on 8 May 2013.

His HCV treatment was discontinued prematurely at week 39 of the intended 48 weeks, due to severe thrombocytopenia. He had close follow-up in the Hepatitis C clinic including complete blood count (CBC) monitoring every 2 weeks. During his last week of therapy, his platelet count decreased from 129,000 to 12,000. Of note, his therapy had been complicated by multiple significant adverse events including severe anemia with a hemoglobin nadir of 7.8, and the development of two abscesses, a suprapubic and a facial abscess. The suprapubic abscess was treated with a course of amoxicillin/clavulanic acid and the facial abscess was treated with clindamycin. Amoxicillin/clavulanic acid may increase the risk of bleeding when given with warfarin but neither antibiotic should significantly affect the INR [4]. His anemia had been difficult to manage as he was unable to tolerate normal dosing ranges of ribavirin. Approximately 7 weeks after starting ribavirin, his dose was reduced to 1000mg/day. Two weeks later, it was reduced again to 600mg/day and eventually down to 200mg/day. He continued to have CBC monitoring by the Hepatitis C clinic with continual ribavirin adjustments. His maintenance dose fluctuated from 200 to 800mg/day, significantly less than his starting dose. Due to his anemia, he required epoetin alfa supplementation throughout his treatment course and was placed on ferrous sulfate and cyanocobalamin. Fortunately, his HCV viral load had been undetectable since week 10 of therapy. He did not experience any bleeding throughout his entire course. Given the severity of his adverse events along with his improved viral load, it was decided to discontinue HCV therapy as overall risks outweighed benefits.

Upon discontinuing boceprevir, his warfarin dose was prophylactically decreased by 17% anticipating that his INR may start increasing. However, at his 1 week follow-up visit, this caused his INR to decrease to 1.48. The warfarin dose was subsequently increased by 10% which resulted, 2 weeks later, in a therapeutic INR of 2.8. Over the following several weeks, his warfarin dose was continuously adjusted. Upon stabilization of his INR, his warfarin dosing needs decreased to 23.75mg/week. This is a 21% reduction from his dose requirement of 26.25mg/week while on boceprevir, and a 37% increase from his original maintenance dose of 15mg/week prior to starting boceprevir.

## Discussion

Boceprevir is a HCV NS3/4A protease inhibitor approved by the US Food and Drug Administration in 2011, for the treatment of chronic HCV genotype 1 infection in combination with peginterferon and ribavirin [3]. Boceprevir is extensively metabolized in the liver, undergoing both oxidation and reduction [3, 4]. Despite a lack of *in vivo* interaction studies between boceprevir and warfarin, the manufacturer suggests warfarin concentrations may be decreased or elevated by boceprevir, and the INR should be monitored closely [3]. *In vitro* studies indicate boceprevir is primarily metabolized by hepatic aldo-keto reductase and partially metabolized by CYP3A4/5, and it is a potent inhibitor of CYP3A4/5 [4, 5]. Other major CYP450 enzymes, such as CYP1A2, 2C8, 2C9, and 2C19, have not been found to be inhibited by boceprevir, although little to no induction has been seen [6].

Warfarin, an oral anticoagulant, consists of a racemic mixture of active enantiomers. Elimination occurs almost exclusively by metabolism through CYP450 isozymes. The S-enantiomer, which is more potent than the R-enantiomer, is predominantly metabolized by the CYP2C9 isozyme, whereas the R-enantiomer is primarily metabolized by CYP1A2 and CYP3A4. Inhibitors and inducers of CYP2C9, CYP1A2, and CYP3A4 are likely to result in increased and decreased concentrations of warfarin, respectively [7]. Because boceprevir is a known inhibitor of CYP3A4, it is expected to increase concentrations of warfarin, although there is a possibility that boceprevir could also induce CYP1A2 and CYP2C9 which would result in reduced concentrations of warfarin [4–6].

Although *in vivo* studies between boceprevir and warfarin have not been reported, other interactions between similar drugs have been documented. In one case report, a patient starting telaprevir, along with ribavirin and peginterferon alfa-2a, required an increase in the weekly warfarin dose by 50% above baseline to re-attain a target INR [8]. Similar to boceprevir, telaprevir has been shown in *in vitro* studies to inhibit the metabolism of other drugs, specifically substrates of CYP3A as well as P-glycoprotein [8]. In both case reports including the one currently being presented, telaprevir and boceprevir have demonstrated an inhibition in warfarin activity, contrary to findings from *in vitro* studies indicating such protease inhibitors act primarily as inhibitors of metabolism resulting in increased pharmacological activity [4, 8].

Administration of human immunodeficiency virus (HIV) protease inhibitors has shown interactions with warfarin similar to those described in this case report between boceprevir and warfarin. HIV protease inhibitors shown to inhibit the activity of warfarin include lopinavir/ritonavir, nelfinavir/ritonavir, and saquinavir [9]. Although these agents are primarily known as CYP450 inhibitors, as in the case of boceprevir, they have demonstrated the ability to decrease warfarin’s activity [9].

At each anticoagulation visit, our patient was assessed for multiple factors that could contribute to variations in INR. These factors included: changes in health, diet, alcohol use, tobacco use, renal function, and liver function. Adherence to prescribed warfarin regimen and changes to medications were also assessed at each visit. He denied any missed doses of warfarin at each of his anticoagulation clinic visits. If he had any potential contributing factors to fluctuations in INR other than the addition of boceprevir, it is documented under “Important notes” in Table 1.

He was newly started with peginterferon alfa-2a and ribavirin 30 days prior to starting boceprevir. A literature review has shown evidence that interferon may potentiate the effect of warfarin, although this was not the effect seen in our case report [10]. In the case of warfarin and ribavirin, a drug interaction search using Micromedex® indicates there are no known drug interactions [11]. Upon literature review, a case report by Schulman reports an inhibition of warfarin activity by ribavirin [12]. In Schulman’s report, the interaction occurred immediately after initiation of the antiviral therapy [12]. However, in our case report, the INR did not decline until 7 weeks after ribavirin was started. Furthermore, the dose of ribavirin was changed frequently in the patient due to his anemia. Despite ongoing ribavirin dose changes, his INR did not change significantly; see Table 1 for details. In another study, ribavirin has been found to induce factor VII messenger ribonucleic acid (mRNA) in patients with hemophilia resulting in a reduction in bleeding episodes [13]. Due to the lack of clear evidence of boceprevir inducing CYP450 enzymes responsible for warfarin metabolism, it is possible that the inhibition of warfarin activity may be due to other unknown causes, such as in the case of ribavirin inducing factor VII mRNA.

Approximately 1 month after starting boceprevir, our patient was also started on epoetin alfa and cyanocobalamin which are not known to interact with warfarin and are unlikely causes for his decrease in INR [7, 11]. Of note, he did consume high vitamin K food beyond his normal intake, prior to his 25 September 2012 visit, at which his INR was subtherapeutic. High vitamin K-rich food can decrease the INR and therefore, may have been a contributing factor to his low INR [7]. However, his INR was also low at his 19 September, 1 and 10 October visits at which times he states he did not consume high vitamin K food. The Naranjo probability scale for this case report indicates a possible relationship between the interaction of boceprevir and warfarin [14]. Ruling out other contributing factors, it appears his trend of subtherapeutic INRs was due to the addition of boceprevir to his medication regimen.

## Conclusions

In this article, we present a case of a patient whose INR decreased significantly after the start of boceprevir. His original warfarin dose was increased by 75% before reaching therapeutic INR 2 months after starting boceprevir. From our literature search, this is the first documented case report regarding this drug interaction. Anticoagulation providers should be aware of this possible interaction and how it can have significant effects on INR and, subsequently, warfarin dosing. Close monitoring is recommended when boceprevir is added to a medication regimen for a patient also on warfarin.

## Consent

Written informed consent was obtained from the patient for publication of this case report. A copy of the written consent is available for review by the Editor-in-Chief of this journal.
